# Accelerating Content-Based Image Retrieval via GPU-Adaptive Index Structure

**DOI:** 10.1155/2014/829059

**Published:** 2014-03-20

**Authors:** Lei Zhu

**Affiliations:** School of Computer Science and Technology, Huazhong University of Science and Technology, Wuhan 430074, China

## Abstract

A tremendous amount of work has been conducted in content-based image retrieval (CBIR) on designing effective index structure to accelerate the retrieval process. Most of them improve the retrieval efficiency via complex index structures, and few take into account the parallel implementation of them on underlying hardware, making the existing index structures suffer from low-degree of parallelism. In this paper, a novel graphics processing unit (GPU) adaptive index structure, termed as plane semantic ball (PSB), is proposed to simultaneously reduce the work of retrieval process and exploit the parallel acceleration of underlying hardware. In PSB, semantics are embedded into the generation of representative pivots and multiple balls are selected to cover more informative reference features. With PSB, the online retrieval of CBIR is factorized into independent components that are implemented on GPU efficiently. Comparative experiments with GPU-based brute force approach demonstrate that the proposed approach can achieve high speedup with little information loss. Furthermore, PSB is compared with the state-of-the-art approach, random ball cover (RBC), on two standard image datasets, Corel 10 K and GIST 1 M. Experimental results show that our approach achieves higher speedup than RBC on the same accuracy level.

## 1. Introduction

With the popularity of high-end digital imaging device and convenient image storage system, the amount of high-quality images is growing exponentially. It provides us a great challenge to find visual similar images from massive image collections. Content-based image retrieval (CBIR) is one of the desirable solutions to return similar images for a given query instance automatically based on pure visual analysis and similarity comparison [1, 2].

CBIR represents the visual content of image with low-level descriptors via feature extraction. With the extracted visual features, similarities between query images and images in database are calculated and sorted, and the most similar image is returned. For a given query image, response time of retrieval depends on the complexity of similarity comparison, which is further determined by the dimension of image feature and image database size. For accurate description, high dimensional feature is needed to represent the complex image content, which is generated from high quality imaging devices. At the same time, large quantities of images are produced from portable image capturing devices. High dimensional features and large quantities of images make the process of similarity calculation and ranking time-consuming. Retrieving similar images becomes a computationally expensive task. For a practical retrieval system design, the efficiency of CBIR system is turned into a crucial issue that needs to be seriously taken into consideration.

To ease the computational pressure, a huge variety of strategies and efforts have been made by researchers in the literature to reduce the retrieval time of CBIR. On the one hand, software developers employ graphics processing unit (GPU) as one of the important platforms to accelerate feature extraction and similarity comparison. Many researchers have reported their promising results on parallelizing sequential algorithm on GPU. Their successful practices demonstrate that hardware acceleration becomes one of the important options to improve the retrieval efficiency. On the other hand, algorithm designers propose many effective index structures at the algorithm design level to accelerate the process of similarity comparison via pruning unnecessary computations. These index structures generally have elegant mathematical formulation but pay little attention to parallel implementation. Oversight of parallel implementation during algorithm design makes the conventional index structures interleave series of similarity calculations and comparisons. In fact, inherent dependence between calculation elements in traditional index structures is not adaptive to capture the characteristics of GPU, which launches a bundle of threads to run independent tasks. Therefore, direct parallelization of traditional index structure cannot make full use of GPU resources. To the best of our knowledge, few papers concentrate their efforts on designing GPU-adaptive index structure to integrate both efforts from algorithm design and hardware parallelization.

In this paper, we are motivated to design our approach via combining the advantages of both algorithm design and hardware parallelization. This is our starting point to develop the GPU-based parallel CBIR system. A novel GPU-adaptive index structure, termed as plane semantic ball (PSB), is proposed to leverage multiple semantic balls to cover retrieval space. It should be noted that this technique is different from traditional index structures that exploit hierarchical space decomposition to prune redundant computations. To improve efficiency further, PSB is designed to represent the involved data structures in the form of matrix, so that the data structures can be processed in parallel by different calculation units in GPU. In this case, large number of threads can be launched on GPU platform to conduct the same operations concurrently, and thus the online retrieval process of CBIR can be greatly improved.

The contributions of the paper are mainly concentrated on the following three facets.

- A novel GPU-adaptive index structure, PSB, is proposed to index the features in database. Different from the existing approaches, PSB is designed to capture the hardware characteristics of GPU.
- An effective parallel strategy is designed to leverage both the space decomposition ability of PSB and the hardware resources of GPU.
- Comprehensive experiments on two image datasets are conducted to demonstrate the effectiveness of the proposed approach.

The remainder of the paper is organized as follows: related work is reviewed in Section 2.  Section 3 provides the background of CBIR system. Data structure and parallel retrieval algorithm are introduced in Section 4.  Section 5 introduces the characteristics of GPU and programming environment CUDA.  Section 6 presents the details of parallel acceleration. Experimental results and discussions are given in Section 7. We finally conclude the paper in Section 8.

## 2. Related Work

This section presents related work on algorithm acceleration and hardware parallelization of CBIR. Related work about algorithm acceleration is mainly concentrated on dimension reduction and index structure. Hardware parallelization is mainly focused on parallel acceleration of CBIR on platform of multicore or many-core.

Dimension reduction is an approach that reduces the dimension of feature vector prior to any application and speeds up the process of similarity computation. In this research field, a huge variety of effective algorithms, such as principal component analysis (PCA) [3], local linear embedding (LLE) [4], and locality preserving projection (LPP) [5], are proposed. The basic idea of these techniques is eliminating the redundant information in feature dimensions and compressing the feature into low dimensions. Although these techniques can accelerate the retrieval process to some degree, they incur some information loss, which negatively impacts the descriptive ability of image feature and brings down the retrieval accuracy.

Index structure is another effective approach, which prunes the retrieval space and avoids unnecessary computations. In general, this kind of approaches can be further divided into two main categories: tree-based index structure and hashing-based index structure. Tree-based index structure performs well for low-dimensional feature (around 20 dimensions), but poorly for high-dimensional feature. The performance of it even degrades to that of brute force retrieval in extreme case when the dimension of features becomes very high. In CBIR, hundreds of feature dimensions are needed to describe the complex visual content of image. Therefore, the retrieval performance will be degraded if tree-based indexing structure is used to index features. Furthermore, tree-based index structure involves an interleaved series of similarity computations and comparison operations, causing ineffectiveness for parallelization [6]. Although hashing-based index structures, such as local sensitive hashing (LSH) [7] and spectral hashing (SH) [8], are proposed without the drawbacks described above, they are not designed for the generic distance types, and thus the application of these algorithms is largely limited. In addition, another weakness of hashing-based index structure is that the building of it involves many parameters, which cannot be easily adjusted for any inexperienced users.

There is also a large body of work focusing on hardware parallelization of retrieval process in CBIR. The authors of [9, 10] propose an effective parallelization of brute force retrieval using compute unified device architecture (CUDA) and CUDA basic linear algebra subroutines (CUBLAS) [11], respectively. An effective algorithm is given in [12] to further extend the process of the nearest neighbor search on multi-GPU. A parallelization of CBIR system is reported in [13] to accelerate the retrieval process on 8-core system and 16-core system. Their experimental results report 5.6x and 9.7x speedups. The work in [14, 15] exploits a computer cluster system to parallelize CBIR with several computers connected by high-speed network. The authors of [16] develop a three-level flexible image retrieval system (FIRE) using open multiprocessing (OpenMP) and shared memory multiprocessors. Challenges, such as load balancing, parallelization overhead, and parallelism exploitation, are considered in their work. All methods described above can only be considered as brute force parallelization, which is inherently parallelizable. Although they have reported promising experimental results, their performance can be further improved if principals of index structure are taken into account.

The approaches that are most similar to this work are [17, 18]. They suggest the nearest neighbor search on GPU via exploiting a simple index structure, termed as a random ball cover (RBC). To the best of our knowledge, RBC may be the most effective GPU-adaptive index structure. However, this work only exploits a simple random subset as representative pivots for space decomposition, which lacks semantics cover for the feature database. In addition, RBC only selects the closest ball as retrieval scope for each query feature, which brings much performance loss. Therefore, in order to reduce accuracy loss, more representative pivots have to be selected to cover more information. Moreover, their approach only focuses on algorithm design and pays little attention to efficient parallel implementation. To mitigate the drawbacks of the RBC described above, we develop a new GPU-adaptive index structure PSB in this paper. In PSB, semantics are embedded to generate representative pivots to make the feature space more compact. Appropriate number of representative pivots is selected according to the similarity measurement to cover more semantic balls and involve more possible reference features. In addition, to maximize the performance of the proposed index structure, we also propose a high-efficient parallel retrieval scheme to make full use of the GPU resources to accelerate the retrieval process.

## 3. Overview of CBIR System

This section reviews the background of CBIR system. For more detailed analysis of relevant techniques, one can refer to [1, 2].

**Figure 1:**
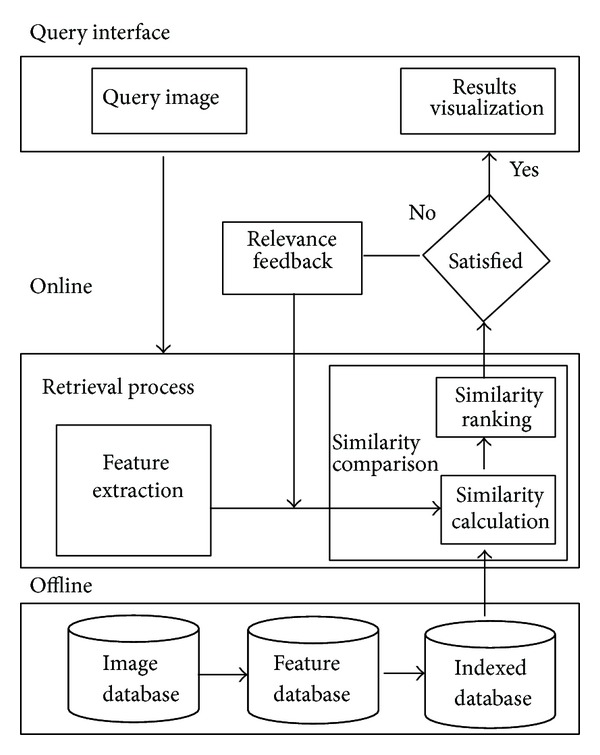
Typical framework of CBIR system.

Although many CBIR systems, such as VisualSeek [19], MARS [20], and SIMPLIcity [21], have been proposed in multimedia field, the majority of them are designed with the same architecture. These systems are generally comprised of an offline part for feature indexing and an online part for query interface.  Figure 1 shows the typical framework of a CBIR system.

In the offline part, visual features of images in database are automatically extracted and stored in feature database. This process is called feature extraction. In computer vision field, many effective approaches are proposed to extract feature descriptors to represent the visual content of image. In general, these features are classified into global features and local features, which represent images by vectors and vector sets, respectively. Since the main aim of this paper is to focus on the acceleration of retrieval process, we simply adopt GIST [22] as the main image feature. The effectiveness of our approach on other features will be explored in further work. In the literature, GIST is proposed to use a set of perceptual dimensions (naturalness, openness, roughness, expansion, and ruggedness) to describe the spatial structure of the image. A Gabor filter with fixed parameters is built. Image is filtered and segmented into grid cells where orientation histograms are extracted. Finally, the response of Gabor filers on each grid cells is concatenated into GIST feature. With extracted GIST features, distances between images are calculated. In practical CBIR system, to speed up the retrieval process, features are usually indexed by data structures to prune the unnecessary computations. An effective index structure should keep a balance between retrieval accuracy and efficiency. The conventional tree-based or hashing-based index structures are proposed to achieve this requirement.

In the online part, given a particular query image, visual feature is extracted as the images in the database. Dissimilarities between query feature and features in database are then calculated and ranked. Based on the ranked results, the corresponding images are finally forwarded to user.

In a typical CBIR system, for the limited discriminating ability of low-level features, several rounds of relevance feedback need to be iterated to bring the semantic gap between low-level features and high-level semantics. In each round of relevance feedback, as descriptive information of query image is revised by the feedback information, similarities between query feature and features in database should be all recalculated. To make things worse, in a practical system, several rounds of feedback are generally needed to achieve query intend, which brings more computational pressure on the underlying hardware.

In general, the stage of similarity calculation and ranking consumes most of the online retrieval time. Hence, it has great importance to develop efficient index structure to reduce the response time for query and improve the user experience for a CBIR system.

## 4. Plane Semantic Ball

In this section, details of the proposed GPU-adaptive index structure PSB and its associated parallel retrieval algorithm are presented. The main notations in the paper are listed in Notations section.

In offline part of CBIR, we build the structure of PSB. Representative pivots are generated from clustering process and balls are built for all pivots to cover the whole feature space. With the built balls, features in database are assigned to their proper balls according to the nearest distance principle. We call the built index structures as semantic since the clustering process embeds semantics into the representative pivots and balls.

With the built PSB, the online retrieval process is divided into two consecutive stages. In the first stage, several nearest representative pivots are selected for query. Features in balls of the selected pivots are combined to constitute the retrieval scope of the second retrieval. The nearest features are found in the second stage and considered as the final results of the whole retrieval system.

### 4.1. Basic Computational Unit

In this subsection, we introduce the module, basic computation unit (BCU), on which both the PSB and online retrieval algorithm are built. The basic aim of BCU is to find  *K*  nearest features for query. We denote the operation of BCU as **N** **N**(**X**, **Y**, *K*), where  **X** is the set of query features,  **Y** is the set of reference features, and *K* is the number of the nearest features which are returned by BCU.

We further decompose the procedure of BCU into two subprocedures: distance computation and distance selection. As the query image *q* is described by a feature vector and feature space is actually vector space, the process of distance computation can be easily transformed into vector-matrix multiplication. This operation can be further transformed into matrix-matrix multiplication when streams of queries are launched. Therefore, many existing GPU implementations of vector-matrix and matrix-matrix multiplication can be applied directly. As image features are generally sparse enough, optimization tricks of sparse matrix-vector multiplication can be further exploited to improve the performance. With BCU presented above, we introduce the construction of our proposed index structure PSB and its associated retrieval algorithm in the following subsections.

### 4.2. PSB Construction

**Figure 2:**
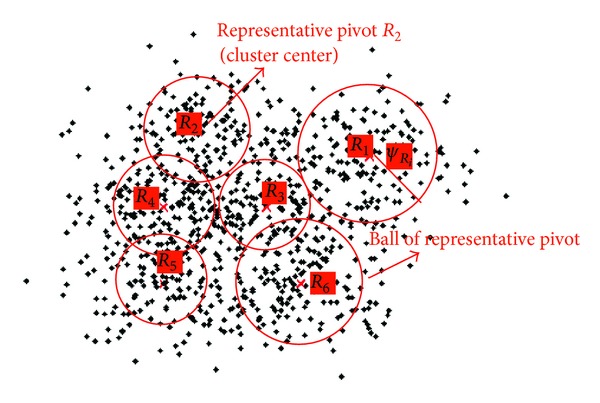
Basic structure of PSB (*R*_1_,* R*_2_,* R*_3_,* R*_4_,* R*_5_,* R*_6_ are representative pivots).

In this subsection, details about the construction of PSB are described. To build PSB, we should first choose representative pivots from feature database  **F** to cover the whole feature space. Each representative pivot maintains a ball which holds *T* nearest features from database. Representative pivots are generated by clustering the feature database to involve more semantics into the cluster centers, which are considered as the representative pivots in this study.  Figure 2 shows the basic structure of our proposed GPU-adaptive index structure PSB.

In PSB, representative pivots are determined by *k*-means. The ball of a particular representative pivot *R*_*f*_ is built from **N** **N**(*R*_*f*_, **F**, *T*) to find the *T* nearest neighbors among  **F**. Its formula can be represented as (|·| is the cardinality of set)

(1) $$\begin{array}{c} \text{Ball}\left(R_{f}\right)=\left\{f\;|\;f\in F,D\left(R_{f},f\right)<\Phi_{R_{f}}\right\},\\ \left|\text{Ball}\left(R_{f}\right)\right|=T,\\ \Phi_{R_{f}}=\underset{f\in \text{Ball}(R_{f})}{\text{MAX}}\left\{D\left(R_{f},f\right)\right\}. \end{array}$$

By properly setting the value of *T*, feature points in database will belong to at least one semantic ball. The combined balls of the *H* nearest representative pivots are defined as **U** **n** **i** **o** **n**(*R*_1_, *R*_2_,…, *R*_*H*_), which is calculated by the** Union** operation on corresponding balls. Formally, it is calculated by **U** **n** **i** **o** **n**(Ball(*R*_1_), Ball(*R*_2_),…, Ball(*R*_*H*_)).

### 4.3. PSB-Based Online Retrieval

Once the user uploads query image, feature is first extracted and image is represented by feature vector. With the PSB in place, the online retrieval process is divided into two consecutive retrieval rounds. The aim of the first round retrieval is reducing retrieval scope, while the second round retrieval is to return the final nearest images. Formally, the first retrieval round can be represented as **N** **N**(**Q**, **R**, *H*), which is used to find *H* nearest representative pivots for each query among *S* representative pivots. The search scope of the second process is determined as the** Union** ball of *H* representative pivots, which can be represented as** Union**(**N** **N**(**Q**, **R**, *H*)). Thus, the whole retrieval process can be represented as** NN**(**Q**, **U** **n** **i** **o** **n**(**N** **N**(**Q**, **R**, *H*)), *E*), which is simply built on the BCU and** Union** operation on balls of the nearest representative pivots. Based on the returned results, the corresponding images are returned to user.

Let us analyse the time complexity of online retrieval. We assume the whole feature database is comprised of *M* images and *N* query images, which are represented by feature vector with *d* dimensions. If brute force retrieval is adopted, the time complexity will be *O*(*MNd*). In our approach, as there are *S* representative pivots and each of them points to *T* image features, the time complexity of online retrieval is *O*(*N*(*S* + *HT*)*d*) ((*S* + *HT*) < *M*). As semantics are embedded in PSB by clustering, small *S* and *H* are required to obtain the same retrieval accuracy with the brute force retrieval. This inference is validated in our experiments which demonstrate that PSB can achieve high speedup with almost no accuracy loss.

## 5. GPU and CUDA

**Figure 3:**
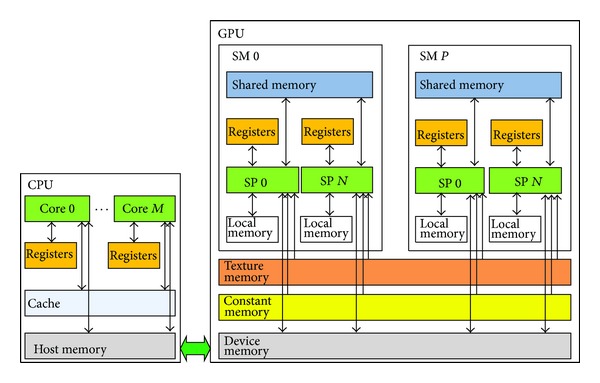
Typical architecture of GPU.

In this section, GPU and its programming environment CUDA are introduced to give a general description. Instructions that should be considered during the parallel implementation are also presented.  Figure 3 shows a typical GPU architecture. GPU came about as a powerful many-core processor for graphic rendering. It usually contains several streaming multiprocessors (SM) which are capable of supporting concurrently threads. A typical SM is generally equipped with multiple streaming processors (SP) and low-capacity shared memory, which has low data access latency and speedy bandwidth. Global memory is the only unique memory type in GPU that can be shared by both CPU and GPU. Therefore, data exchange between GPU and CPU is mainly through global memory. The main drawback of global memory is data access latency caused by PCI transmission. In addition, GPU is equipped with constant and texture memory to speed up the process of read/write operation for frequently accessed data.

In order to enable the general applications to run on GPU, we develop a CUDA C-like program that parallelizes the sequential algorithm. To program with CUDA, sequential algorithms should be first analyzed and decomposed into sequential and parallel programs, which can be dispatched to run on CPU and GPU, respectively. Parallel programs are organized into kernels, which launch threads to run the same code on segmented data concurrently.

Architecture characteristics of GPU and special programming mode of CUDA present significant challenges for parallel algorithm design. To maximize the efficiency, sequential algorithms should be seriously analyzed and decomposed into components with low dependencies. In addition, the characteristics of parallel programming and memory optimization tricks should be carefully taken into account.

## 6. Parallel Acceleration

### 6.1. Data Layouts

A GPU-based parallel algorithm that is memory bound may, as is the case in CBIR, yield poor performance if the programmer ignores the specific characteristics of GPU. Therefore, to obtain better memory throughput, data structures included in PSB should be placed in appropriate memory type, by taking into account both the intrinsic GPU memory characteristics and inherent attributes of memory access to data structure.

Since representative pivots and reference features are almost constant in the whole retrieval process, they are placed on constant memory and texture memory, respectively, to improve memory throughput. For large-scale image dataset, reference features should be placed on global memory as the capacity of texture memory is limited. Because query features are varied, these features are directly transferred to global memory to provide memory coalescing. Note that all features described above are transformed in columns so that each thread can access one component of current query data.

### 6.2. Parallelization of BCU

Since the retrieval process of queries is independent, we parallelize the computation of BCU on query level. It means that the retrieval processes of queries are parallelized.

#### 6.2.1. Distance Matrix Computation

The aim of this step is to calculate the pairwise distance between queries and reference features, generating distance matrix *D*. The element in *i*th row and *j*th column of matrix is denoted as *D*_*ij*_, which represents the distance between query feature *i* and reference feature *j*. For presentation convenience, in our approach, the distance metric considered between two image features is trivially restricted to Euclidean distance. This metric can be naturally substituted with other metrics for other features. Formally, *D*_*ij*_ is given by

(2) $$\begin{array}{c} D_{ij}=\sqrt{\sum_{l=1}^{d}{\left(X_{lj}-Y_{li}\right)}^{2}.} \end{array}$$

Matrix multiplication can be implemented on GPU efficiently since it can be decomposed into many independent calculation elements. Therefore, in order to make full use of the GPU resources, the computation of distance matrix is transformed into matrix-based calculation. Formally, the value of *D*_*ij*_ can be calculated as

(3) $$\begin{array}{c} D_{ij}=\sqrt{{\left(X_{j}-Y_{i}\right)}^{T}\left(X_{j}-Y_{i}\right)},\\ X_{j}={\left(X_{1j},X_{2j},\ldots ,X_{dj}\right)}^{T},\\ Y_{i}={\left(Y_{1i},Y_{2i},\ldots ,Y_{di}\right)}^{T}. \end{array}$$

After mathematical transformation, *D* can be calculated as

(4) $$\begin{array}{c} D=\sqrt{A+B+C}, \end{array}$$

where the *i*th row of *A* is the modulus of *Y*_*i*_ and denoted as ||*Y*_*i*_|| and the *j*th column of *B* is the modulus of *X*_*j*_ and denoted as ||*X*_*i*_|| (||·|| is the Euclidean norm). The matrix *C* is computed in form of the matrix-matrix multiplication between query and reference features as

(5) $$\begin{array}{c} A_{ij}=||Y_{i}||=\sqrt{\sum_{l=1}^{d}Y_{li}^{2}}B_{ij}=||X_{j}||=\sqrt{\sum_{l=1}^{d}X_{lj}^{2}},\\ C_{ij}=-2\times \sum_{l=1}^{d}X_{lj}\times Y_{li}D_{ij}=\sqrt{A_{ij}+B_{ij}+C_{ij}}. \end{array}$$

Since, in the context of CBIR, we only need the rank order of reference features in two retrieval rounds, the values of distance are not valuable. Therefore, the matrix calculation of *B* is omitted for simplicity:

(6) $$\begin{array}{c} D=\sqrt{A+C}. \end{array}$$

The computation of matrix *C* is matrix-matrix multiplication. There are a lot of existing implementations. Therefore, we do not implement our own GPU code of matrix-matrix multiplication, but just use the optimized implementation in CUBLAS. In this way, to compute the matrix *D*, we just calculate the modulus of reference features and add it to proper position in *C*. In our approach, this process is completed in coarse interreference parallelism. Threads are launched with the same number as reference features. Each thread calculates the modulus and adds the value to the corresponding column.

#### 6.2.2. Distance Selection

To this end, distances between query features and reference features are computed. This step is to select the nearest distances for queries. Two available strategies can be adopted to complete this process. The first strategy that can be taken is to select minimum values directly on distances. Its main drawbacks are the memory abusing to store the entire distance vector in global memory and the time delay as a result of continuous memory access.

**Figure 4:**
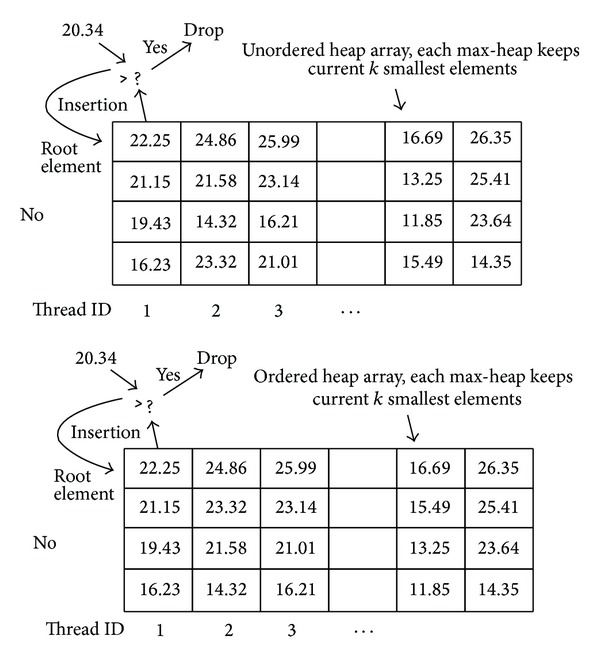
Two types of heap-array built for features of query images (ordered heap keeps the stored minimum elements ordered, but unordered heap does not need this requirement).

In our implementation, a heap-array is built for features of query images, which means that a max-heap (root element is largest one is Heap) is built for each query. Two types of max-heap are built in retrieval process. In the first retrieval round, an unordered max-heap is built to store the distances of unsorted representative pivots. In the second retrieval round, ordered max-heap is built to store the distances of final nearest reference features.  Figure 4 shows the basic structure of ordered and unordered heap-array built in our approach. The only difference of two types of max-heap is that whether the stored indexes of nearest features are ordered or not.

Max-heap keeps track of elements with minimum distance that have been found so far and excludes unnecessary distance comparisons. We assume to find *K* smallest distances (in the first retrieval round *K* = *H*, in the second retrieval round *K* = *E*). In the beginning, first *K* distances are inserted into the heap directly without comparison. When the heap is full, the newly encountered elements are inserted with comparison. Unnecessary distance comparisons can be excluded directly by comparing the input value with root counterpart in max-heap. If the value of newly encountered element is larger than root element, it is excluded directly. In contrast, if the value is smaller than root element, it is pushed into its corresponding position in distance heap after adjustment. For ordered max-heap, indexes of stored features should always be kept ordered after each adjustment.

After the insertion of elements, *K* smallest distances are obtained. However, the main aim of distance selection is to obtain the indexes of the corresponding reference features, but not the distance values. To accomplish this goal, an index Heap is allocated as a counterpart to store the indexes of nearest reference features. The element insertion operations performed in distance Heap are applied to index Heap again. After several rounds of comparison and insertion, the indexes of nearest reference features for query features are obtained and are finally stored into the global memory.

In order to take advantages of both max-heap and inherent characteristics of GPU memory types, coarse interquery parallelism is exploited for parallelization of this procedure. A kernel function is designed and threads are launched with the same number as queries. These threads compute distance between queries and reference features and select indexes of *K* minimum distances from the distance vector.

### 6.3. Parallel Retrieval

**Figure 5:**
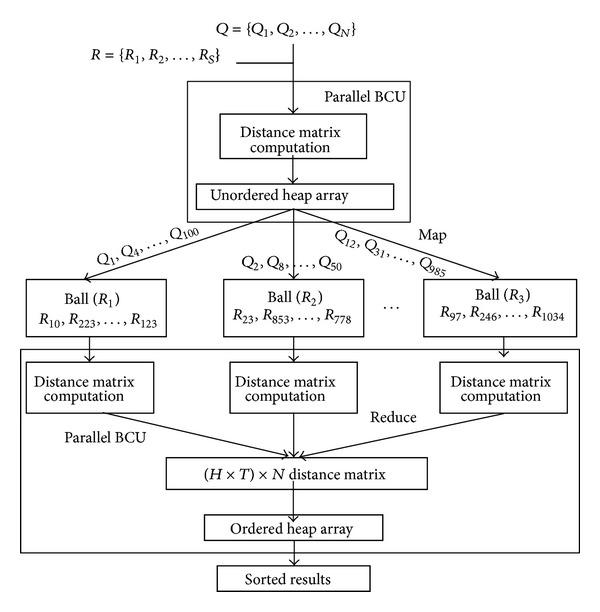
Parallel online retrieval in CBIR.

Based on the parallel BCU, a MapReduce-like parallel retrieval scheme is designed for CBIR.  Figure 5 describes the basic framework of our parallel retrieval process of CBIR. In this framework, features of query images and that of representative pivots are first imported into the module of parallel BCU where unordered heap array is already built. After that, *H* nearest representative pivots are found for each query feature. Queries are then mapped to their corresponding semantic ball, where distance matrix is computed between queries and reference features. A reduce operation is designed to collect the distances calculated for each query feature from *H* balls. This procedure generates (*HT*) × *N* distance matrix, which is further imported into the ordered heap array. After these procedures, indexes of the nearest reference features are obtained and their corresponding images are returned to query user.

## 7. Experiments and Results

In this section, a set of experiments are performed to demonstrate the performance of the proposed methodology. First, details about the test dataset and experimental setup are introduced. Second, performance variation is investigated with parameters of our approach. Third, our approach is compared with brute force retrieval and the state-of-the-art approach on Corel 10 K [23], respectively. Finally, comparative experiment is performed on GIST 1 M [24] to evaluate the performance on large-scale image dataset.

### 7.1. Dataset and Experimental Setup

The proposed approach is evaluated on standard image dataset Corel 10 K and GIST 1 M, which are widely used in the literature for CBIR performance evaluation. Corel 10 K contains 10000 images in 100 categories. In experiment, 10 images for each category are randomly selected as query images. The remaining images are used as reference images to be retrieved. For Corel 10 K, GIST is extracted with 4 blocks and 8 orientations, generating 512-dimensional features. GIST 1 M dataset has two feature datasets: query and reference dataset. The reference dataset contains features of the Holidays image set and images from Flickr 1 M [25]. The query dataset only contains features from the Holidays image. On GIST 1 M dataset, all images are represented by 960 feature dimensions. Statistics of dataset is given in Table 1.

In experiments, GPU code is implemented using CUDA. All the experiments are performed on the platform equipped with GPU of NVIDIA Geforce GTX460, whose CUDA capability is 2.1, drive version is 4.0, and GPU clock rate is 1.55 GHz. The platform is also equipped with an Intel Core i7 950 CPU running at 3.07 GHz. The operating system is 64-bit RHEL AS 6 with Linux kernel 2.6.32.

The performance is evaluated on mean average precision (MAP) and speedup. For Corel 10 K, MAP is calculated as the mean of precisions achieved at all recall levels. For GIST 1 M dataset, MAP is calculated as the mean of precisions when 100 images are returned. Speedup of an approach is calculated as the ratio between run time of approach and that of GPU-based brute force implementation.

### 7.2. Evaluation for Number of Representative Pivots and Radius of Ball

**Figure 6:**
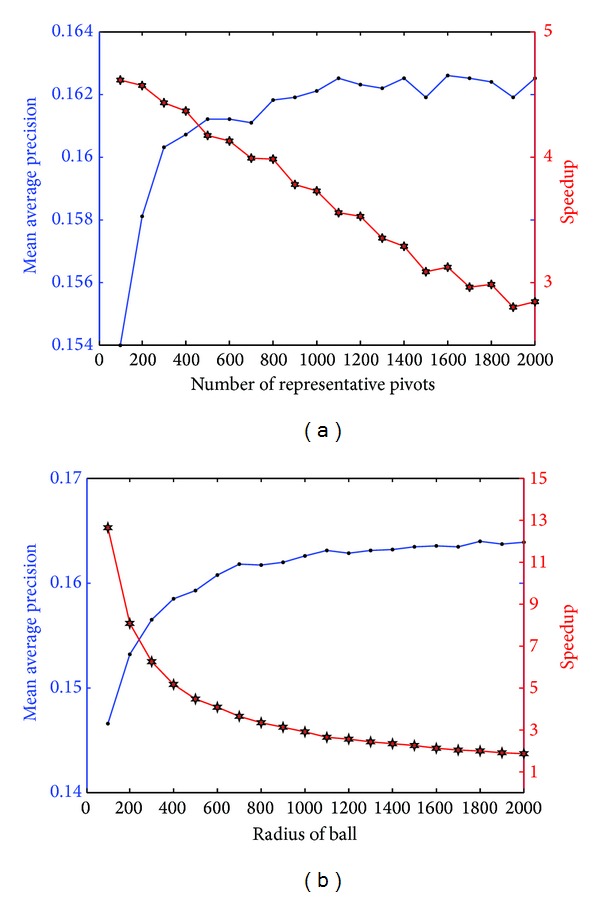
Performance evaluation on Corel 10 K for a number of representative pivots and radius of ball. (a) MAP variations with the number of representative pivots. (b) MAP variations with radius of ball.

Adjusting parameters for PSB keeps a balance between speedup and accuracy loss. In this subsection, experiments are performed to observe the MAP variations with the parameters of PSB.  Figure 6 reports MAP and speedup variations with the number of representative pivots (*S*) and the radius of ball (*T*). For more data, one can refer to Table 2 and Table 3 for details. The figure shows that when parameters are above certain value (*S* = 800 or *T* = 700), MAP curve becomes steady. This experimental phenomenon gives us a revelation that the best parameters of PSB can be determined as the certain point in growth curve of accuracy. In addition, Figure 6 also shows that the proposed PSB can achieve more than 1x speedup with little accuracy loss. For instance, when *S* is 300 and *T* is 1800, the MAP we can obtain is 0.164 (0.0002 smaller than accurate MAP). At the same time, the speedup we get is 1.96, which is almost 2x faster compared with GPU-based brute force retrieval.

### 7.3. Evaluation for Semantic Embedding and Number of Searched Representative Pivot Selection

**Figure 7:**
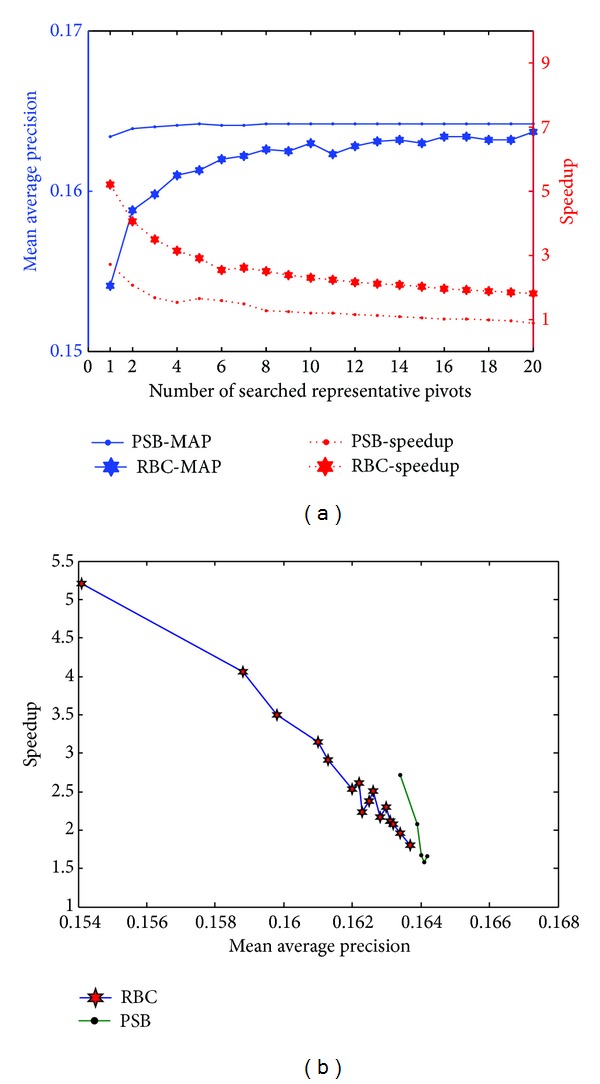
Performance evaluation on Corel 10 K for semantic embedding and multiple representative pivots selection. (a) MAP and speedup variations with the number of representative pivots. (b) Speedup variations with MAP.

In this subsection, we evaluate the effectiveness of semantic embedding and multiple representative pivot selection. In this experiment, *S* and *T* are fixed to 800 and 700, respectively, to observe performance variations.  Figure 7 shows the performance improvement. For concrete data, one can refer to Table 4.  Figure 7(a) shows that the proposed PSB can get higher MAP compared with RBC on each *H*. When the *H* is 5, we can obtain the same MAP with brute retrieval and 1.65x speedup. It clearly demonstrates that the semantic embedding can involve more semantics into the generation of representative pivots and condense the retrieval space. Based on the condensed search scope, higher MAP is easily achieved if the same *H* is set.  Figure 7(a) also reports the MAP and speedup variations with *H*. It shows that MAP of RBC and PSB both increase with *H*. This experimental result shows that multiple representative pivot selection can cover more informative reference features in database. The negative effect of multiple representative pivot selection is that the retrieval process is slightly extended, which makes the speedup decrease steadily with *H*.

From Figure 7(a), we can draw a conclusion that MAP and speedup are varied with *H*. Therefore, for comparison convenience and better presentation, Figure 7(b) is plotted to reflect the relationship between speedup and MAP. This figure clearly illustrates that our approach achieves lower speedup compared with RBC when different *H* is set. But, on the same MAP level, we can achieve higher speedup. In addition, we can obtain the MAP that cannot be achieved by RBC (the best MAP obtained by RBC is 0.1637, while that obtained by PSB is 0.1642).

### 7.4. Comparison with Brute Force Retrieval

In this subsection, we compare the proposed PSB with the brute force retrieval. We directly download the source code of GPU-based brute force retrieval from [10] and use it to find the nearest referenced features for each query. A set of experiments is performed to observe the speedup variations with accuracy loss.

**Figure 8:**
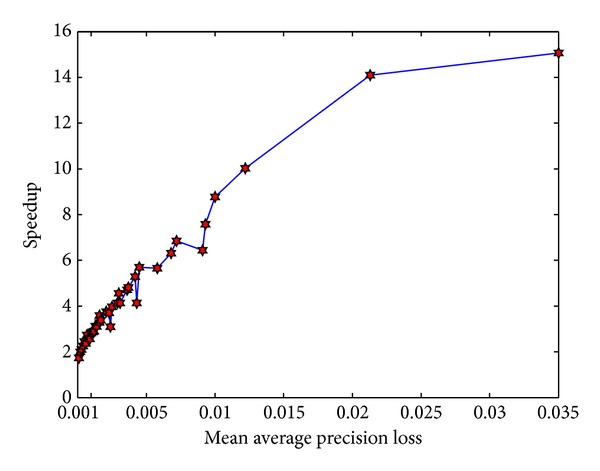
Performance comparison with GPU-based brute force retrieval.

Figure 8 shows that the MAP loss of PSB is generally ranged from 0.035 to 0.0001. The speedup achieved in this context is very important. Even when the accuracy loss is 0.0001 (very close to accurate retrieval), we can obtain 1.73x speedup, 73% improvement compared against brute force retrieval. In addition, on other accuracy loss level, we can easily obtain higher speedup. For instance, we can achieve about 2.8x speedup when the accuracy loss is 0.001 and 3.7x speedup when accuracy loss is 0.002. This experiment clearly demonstrates that we can obtain high speedup with little accuracy loss. It also indicates that the proposed approach is effective in scenario of approximate CBIR.

### 7.5. Comparison with the State-of-the-Art Approach RBC

In this subsection, the performance of PSB is compared with the state-of-the-art approach RBC. Source code of RBC implementation is directly downloaded from author website [26]. In this experiment, parameters of the two approaches are adjusted manually and a figure is drawn to reflect the relationship between speedup and accuracy.

**Figure 9:**
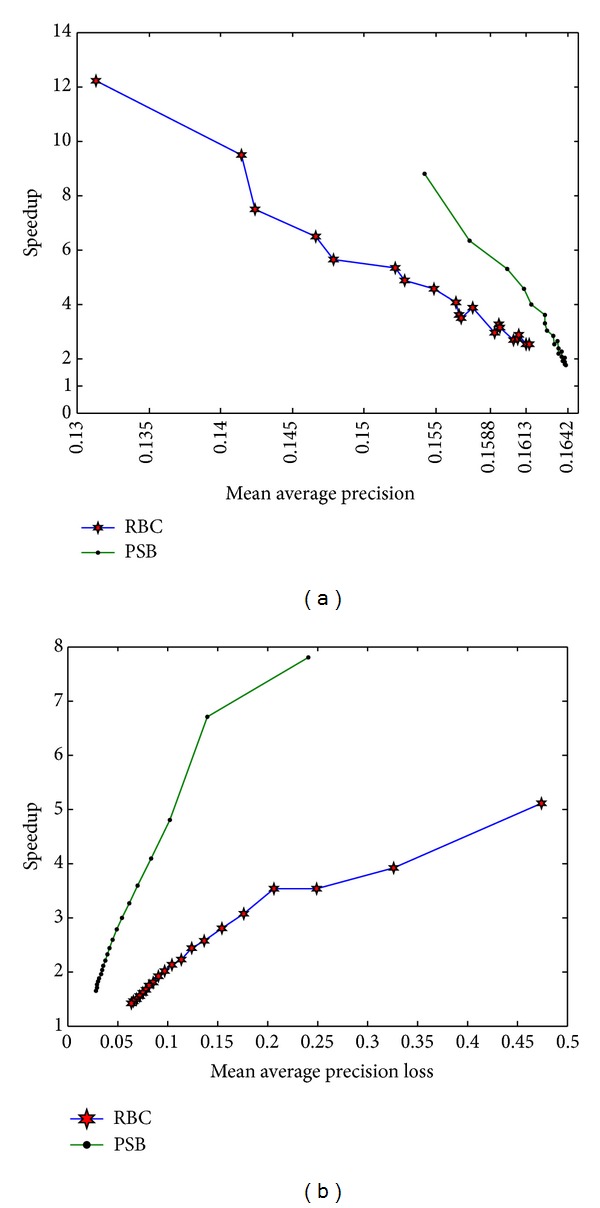
Comparisons with the state-of-the-art approach RBC. (a) Comparison on Corel 10 K. (b) Comparison on GIST 1 M.

Figure 9(a) describes speedup variations of two approaches with MAP on Corel 10 K. We can easily see from the figure that PSB performs better than RBC. The figure shows that PSB can obtain MAP which cannot be reached by RBC. MAP of PSB is mainly ranged from 0.1542 to 0.1641, while that of RBC is concentrated in a range from 0.1313 to 0.1615. The largest MAP PSB obtained is 0.1641, while that obtained by RBC is only 0.1615. More importantly, PSB obtains higher speedup than RBC on the same accuracy level. For instance, when MAP is 0.1613, PSB achieves speedup up to 4.55x, which is larger than 2.51x speedup achieved by RBC. When MAP is 0.1588, speedup of PSB is up to 4.58x, which is also large than 2.82x achieved by the RBC. It should be noted that speedup of PSB is almost 2 times the speedup of RBC when accuracy is about 0.154. The main reason that PSB obtains the better performance is that PSB covers the whole reference features with semantics so that retrieval scope can be reduced.

Figure 9(b) reports speedup variations of two approaches with MAP loss on GIST 1 M. It is not hard to find from the figure that, even with high dimensional features and large-scale image dataset, PSB still achieves better performance than RBC. On this dataset, PSB can obtain MAP loss that cannot be reached by RBC. On the same accuracy loss level, our approach can also achieve higher speedup.

## 8. Conclusions

CBIR is one of the available solutions to find similar images automatically for query from large quantities of images. To overcome the computational complexity of CBIR, a novel GPU-adaptive index structure is proposed in this paper to simultaneously prune unnecessary computations and leverage the parallel processing capability of GPU. With the built GPU-adaptive index structure, the retrieval process of CBIR can be decomposed into independent components that can be parallelized with high efficiency. Effective parallel techniques are designed to make full use of the computational resources of underlying hardware. Experiments on standard image retrieval datasets demonstrate that the proposed approach achieves substantial speedup with little accuracy loss and also achieves higher speedup compared with the state-of-the-art approach. All experiments demonstrate that the proposed approach can be an effective and robust tool to help solve the computational challenges of CBIR. In the future, we will continue to deploy the proposed algorithm to other applications and evaluate its performance.

## Figures and Tables

**Table 1:** Statistics of test dataset.

Image database	Corel 10 K	GIST 1 M
Feature dimensionality	512	960
Image database size	10,000	1,001,000
Query dataset size	1,000	1,000
Reference dataset size	9,000	1,000,000

**Table 2:** MAP and speedup variations with *S* on Corel 10 K (*H* = 3, *T* = 500).

*S*	400	600	800	1000	1200	1400	1600	1800
MAP	0.1607	0.1612	0.1618	0.1621	0.1623	0.1625	0.1626	0.1624
Speedup	4.36x	4.12x	3.97x	3.72x	3.52x	3.28x	3.12x	2.98x

**Table 3:** MAP and speedup variations with *T* on Corel 10 K (*H* = 3, *S* = 300).

T	400	600	800	1000	1200	1400	1600	1800
MAP	0.1585	0.1607	0.1617	0.1626	0.1628	0.1632	0.1635	0.164
Speedup	5.14x	4.07x	3.33x	2.88x	2.54x	2.31x	2.12x	1.96x

**Table 4:** MAP and speedup variations with *H* on Corel 10 K (*S* = 800, *T* = 700).

H	4	5	8	10	12	14	16
RBC-MAP	0.161	0.162	0.1626	0.163	0.1628	0.1632	0.1634
RBC-speedup	3.14x	2.53x	2.50x	2.29x	2.16x	2.07x	1.96x
PSB-MAP	0.1641	0.1642	0.1642	0.1642	0.1642	0.1642	0.1642
PSB-speedup	1.53x	1.65x	1.27x	1.20x	1.15x	1.08x	1.01x

## Notations

*M*: Number of images in database
*N*: Number of query images
*S*: Number of representative pivots
*T*: Radius of ball
*E*: Number of results returned by CBIR
*H*: Number of searched representative pivots
*d*: Dimension of feature vector
*D*: Distance matrix between features
**D**: Dissimilarity measure
*R*_*f*_: Feature of a particular representative pivot
**R** = {*e*_1_, *e*_2_, …, *e*_*S*_}: Feature of a representative pivot
**F** = {*f*_1_, *f*_2_, …, *f*_*M*_}: Feature database
**Q** = {*q*_1_, *q*_2_, …, *q*_*N*_}: Query features.
